# The “Gate Keeper” Role of Trp222 Determines the Enantiopreference of Diketoreductase toward 2-Chloro-1-Phenylethanone

**DOI:** 10.1371/journal.pone.0103792

**Published:** 2014-07-29

**Authors:** Hairong Ma, Xin Yang, Zhuo Lu, Nan Liu, Yijun Chen

**Affiliations:** State Key Laboratory of Natural Medicines and Laboratory of Chemical Biology, China Pharmaceutical University, Nanjing, Jiangsu Province, People's Republic of China; Institute of Enzymology of the Hungarian Academy of Science, Hungary

## Abstract

Trp222 of diketoreductase (DKR), an enzyme responsible for reducing a variety of ketones to chiral alcohols, is located at the hydrophobic dimeric interface of the C-terminus. Single substitutions at DKR Trp222 with either canonical (Val, Leu, Met, Phe and Tyr) or unnatural amino acids (UAAs) (4-cyano-L-phenylalanine, 4-methoxy-L-phenylalanine, 4-phenyl-L-phenyalanine, *O*-*tert*-butyl-L-tyrosine) inverts the enantiotope preference of the enzyme toward 2-chloro-1-phenylethanone with close side chain correlation. Analyses of enzyme activity, substrate affinity and ternary structure of the mutants revealed that substitution at Trp222 causes a notable change in the overall enzyme structure, and specifically in the entrance tunnel to the active center. The size of residue 222 in DKR is vital to its enantiotope preference. Trp222 serves as a “gate keeper” to control the direction of substrate entry into the active center. Consequently, opposite substrate-binding orientations produce respective alcohol enantiomers.

## Introduction

Enantioselectivity is an intriguing property that allows certain enzymes to be exploited to yield enantiomerically pure chemicals for use in diagnostics, materials and pharmaceuticals [1]–[2]. Since enzymes do not possess perfect enantioselectivity when an unnatural substrate is transformed, altering the enantioselectivity of enzymes by protein engineering is a useful biotechnological approach to generate versatile biocatalysts for various enzymatic reactions, and allows analysis of structure-function relationships [3].

Directed evolution is the most promising approach to fine-tune enzyme enantioselectivity to a desired level [4]–[5]. Enantioselectivity inversion is a useful strategy, which can be achieved by either directed evolution or rational design that involves considerable effort. Successful examples of enantiotope preference inversion are available for oxidases, reductases, transaminases and dehydrogenases [6]–[9]. Enantioselectivity inversion often requires changes in multiple amino acid residues, although multi-site mutations can lead to obvious changes in structural features, especially in the active center involved in substrate binding and catalysis. However, single-site mutations can be partially sufficient for inverting the enantioselectivity of certain enzymes, suggesting that enantiopreference is inherent to specific residues.

**Figure 1:**
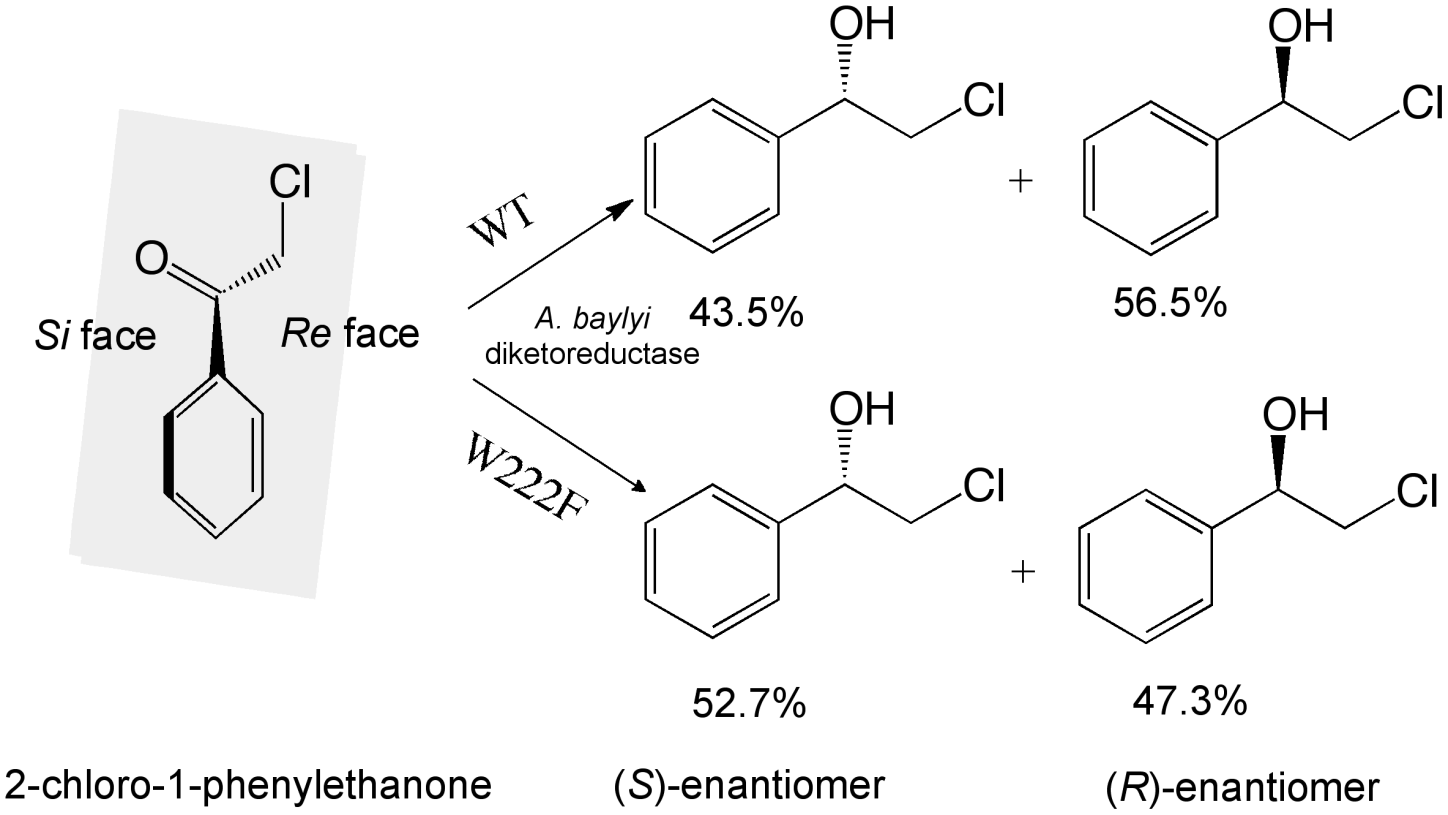
Reduction of 2-chloro-1-phenylethanone to two enantiomeric alcohols by WT-DKR and mutant W222F. WT-DKR display *Re* face preference for production of *R*-alcohol, whereas W222F favors the *Si* face of the ketone.

Diketoreductase (DKR), a homodimeric protein containing 283 amino acids in each subunit, is a useful biocatalyst that stereoselectively reduces β,δ-diketo esters to corresponding dihydroxy products for biosynthesis of statin side chains [10]–[14]. Additionally, DKR reduces a variety of monoketones to chiral alcohols with varying enantiotope selectivity [15]. When 2-chloro-1-phenylethanone is the substrate for this enzyme, the product 1-hydroxy-2-chloro-phenylethane exhibits an *R*-preference. In screening different mutants against the mono-ketone substrate of 2-chloro-1-phenylethanone, enantioselectivity of the W222F variant changed from an *Re*- to *Si*-preference (Figure 1). This result indicates that a mutation at residue 222 mutation inverts the enantiopreference of DKR for this particular substrate.

**Figure 2:**
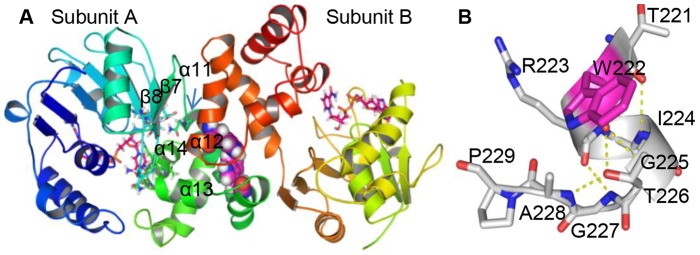
Crystal structure and substrate binding modes of the WT-DKR-NAD^+^ complex. (A) α-helices 11, 13 and 14, and β-strands 7, 8 in subunit A, and α-helix 12 in subunit B form the hydrophobic pocket. Hydrophobic residues located at the active site are shown as sticks with the same ribbon color. Trp222 is shown as a sphere in magenta. NAD is shown as a stick in red. (B) Electrostatic interaction between residues located at the α-helix 12 terminus.

According to a recently solved crystal structure and elucidated catalytic mechanism of DKR [16], two Trp residues at positions 149 and 222 appear to be important for substrate-binding. Indeed, site-directed mutagenesis of these Trp residues revealed their essential roles in maintaining structural integrity and catalytic function [17]. According to our previous study, Trp222 lies at the hydrophobic dimeric interface of DKR (Figure 2A), but does not directly participate in the interaction between the enzyme and substrate (Figure 2B). We thus hypothesized that the size of the Trp222 side chain size plays a critical role in determining DKR enantiotope selectivity. In this study, we substituted Trp 222 with amino acids of varying sizes through conventional mutagenesis and also incorporated unnatural amino acids (UAAs) through genetic code modulations. We found that residue 222 size correlates with DKR enantiopreference toward the ketone substrate 2-chloro-1-phenylethanone. Additionally, residue 222 serves as a “gate keeper” to control the direction of the substrate entrance to the active center with different substrate-binding orientations resulting in the formation of opposite alcohol enantiomers.

## Materials and Methods

### Materials

Primers used in site-directed mutagenesis were synthesized by Invitrogen Inc. (Shanghai, China). Mutations were confirmed by DNA sequencing with an ABI Genetic Analyzer 3730 (Invitrogen Inc., Shanghai, China). *Escherichia coli* strains DH5α and BL21 (DE3) were obtained from Tiangen Biotech Co., Ltd. (Beijing, China). The AxyPrep Plasmid Miniprep Kit was from Axygen Biotech Ltd. (USA). The pEVOL-ONBYRS plasmid was a gift from Professor Peter. G. Schultz at Scripps Research Institute (La Jolla, CA USA). The UAAs 4-cyano-l-phenylalanine, 4-methoxy-l-phenylalanine, 4-phenyl-L-phenyalanine and *O*-*tert*-butyl-l-tyrosine were purchased from Adamas Reagent Co., Ltd. (Switzerland). Isopropyl-β-d-thiogalactopyranoside (IPTG), NADH, and acetoacetyl-CoA were purchased from Sigma Chemical Co. (St. Louis, USA).

### Plasmid construction

The pET22b(+)-DKR construct containing plasmid pET22b and full-length WT-DKR was used as a template for mutagenesis. Site-directed mutagenesis was performed with the QuikChange Lightning site-directed mutagenesis kit (Stratagene, USA) according to the manufacturer's protocol. Two overlapping complementary primers containing the desired nucleotide changes were designed for each mutation reaction. The plasmid pET22b-DKR222TAG with Trp222 mutated to TAG and a stop codon (TGA) was used to incorporate UAAs.

The *p*-cyanophenylalanine specific aminoacyl-tRNA synthetase (pCNFRS) [18] genes containing *Bgl*II & *Sac*I sites were synthesized by Invitrogen Inc. (Shanghai, China). The primer MGU110 (5′- CATATGTAACGCCGTTATACGTTGTT -3′) and MGU111 (5′- GACGTCAAAAGCACGCAAACTCAATA -3′) were used to amplify the *p*CNFRS gene containing *Nde*I and *Pst*I sites at the N-terminus and C-terminus, respectively. These two genes were then digested with restriction enzymes *Nde*I & *Pst*I, and *Bgl*II & *Sal*I, respectively, and then ligated into the plasmid pEVOL in which the ONBYRS gene was deleted after digestion with *Nde*I and *Pst*I. The resulting plasmid contained pCNFRSII-tRNA with two copies of pCNFRS (expressed under the control of an *araBAD* promoter) and a copy of suppressor tRNA (expressed under the control of an *Ipp* promoter). ONBYRS and CNFRS are largely homologous, with differences at only nine amino acid residues (positions 32, 65, 108, 109, 158, 159, 162, 263 and 286), and thus recognize the same cognate tRNA.

### Protein expression and purification

WT-DKR was expressed as described by Wu *et.al.* [15]. To express DKR mutants carrying UAAs, *E. coli* BL21 (DE3) cells were co-transformed with pCNFRSII-tRNA and pET-DKR222TAG. The transformed cells were recovered in 1 ml of LB medium shaken for 1 h at 37°C before plating on a LB agar plate containing chloramphenicol (Cm, 34 µg·ml^−1^) and ampicillin (Amp, 100 µg·ml^−1^). An abridged method for expressing UAA mutants was adapted from a previous report [19]. Briefly, a single colony was inoculated into 100 ml culture and incubated at 37°C overnight with shaking. The cells were then harvested and resuspended in 300 ml M9 media supplemented with 34 µg·ml^−1^ Cm and 100 µg·ml^−1^ Amp. Cells were grown at 37°C with shaking at 220 r.p.m. When the OD_600_ reached 0.5, expression was induced by addition of 1 mM IPTG and 0.2% arabinose for 30 h.

The procedures for purification of DKR and DKR mutants were described previously [14], [20]. Briefly, proteins were purified on HiTrap DEAE FF and Sephadex G-100 columns (GE Healthcare Biosciences, USA) with an ÄKTA purifier 900 (GE Healthcare Biosciences, USA). Purified proteins were examined on 12% SDS-PAGE gels with Coomassie blue staining. Protein concentration was determined by the BCA method (CoWin Biotech Co. Ltd., Beijing, China).

### Chiral HPLC analysis of alcohol products

Chiral HPLC was performed on a Chiralcel OD-RH column (5 µm, 150×4.6 mm) at 25°C with an injection volume of 10 µl and a flow rate of 0.5 ml·min^−1^. Mobile phases A and B consisted of 0.1% trifluoroacetic acid in water and 0.1% trifluoroacetic acid in acetonitrile, respectively. Elution was achieved with a gradient of 25–30% B in 25 min, and kept at 30% B for an additional 5 min. The retention times of (*R*)-1-hydroxy-2-chloro-phenylethane and (*S*)-1-hydroxy-2-chloro-phenylethane were 10.4 min and 12.2 min, respectively.

### Biochemical analysis

The enzyme activity of wild type and mutant DKR was determined on a UV-1700 array spectrophotometer (Shimadzu, Kyoto, Japan) by monitoring the decrease in absorbance of NADH (ε = 6.21 mM^−1^ cm^−1^), as described by Huang *et al.* [21]. Briefly, a standard assay mixture containing 0.1 M potassium phosphate buffer (pH 6.0), 150 µM NADH, 250 µM 2-chloro-1-phenylethanone and 2 µg purified enzyme was prepared. One unit of DKR activity was defined as the oxidation of one µmole NADH per minute per milligram protein.

For kinetic analysis, nine substrate concentrations (0.10, 0.15, 0.20, 0.30, 0.40, 0.50, 0.60, 0.75 and 1.0 mM) or different NADH quantities (0.03, 0.06, 0.09, 0.12, 0.15, 0.18, 0.24 and 0.30 mM) were prepared in the standard assay conditions. *K_m_* and *K_cat_* values were calculated from three independent experiments by a nonlinear regression and plotted using the Michaelis-Menten equation.

Fluorescent spectra were recorded at room temperature on a Tecan Flurospectrometer (Salzburg, Austria) with a Safire microplate reader in combination with XFLUOR4 software, version 4.5. The excitation and emission monochromators were set at 5 nm slit widths. After excitation at 290 nm for selective tryptophan excitement, sample emission was measured from 300 to 400 nm. The average of three separate scans was analyzed, and background due to buffer was subtracted. Because the decrease in fluorescence was attributed to the binding between substrate or NADH and DKR, dissociation constants (K_d_) for substrate and NADH were determined by fluorescence quenching experiments in 96-well plates. DKR was titrated with increasing amounts of substrate/NADH and the resulting decrease in protein fluorescence intensity was monitored in a volume of 200 µl/well containing 0.1M Tris-HCl (pH 6.5), 6.3–7.0 µM enzyme and different final concentrations of the substrate (0–1000 µM)/NADH(0–300 µM). K_d_ values were calculated by fitting the data to the quadratic equation [22], 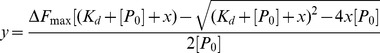 where x (µM) is the ligand concentration, y is the subtraction of the fluorescence intensities in the absence and presence of the ligand (Δ*F*), and [*P*_0_] (µM) is the enzyme concentration.

### Molecular modeling and docking

Structural models of DKR mutants with Val, Leu, Met, Phe, or Tyr at residue 222 in complex with NAD were generated by homology modeling using WT-DKR structures (PDB codes 4E12, 4E13) [16] as templates. Models where residue 222 was replaced with UAAs were generated by the Molecular Builder tool in the Molecular Operating Environment (MOE2009; Chemical Computing Group Inc., Montreal, Canada). All models were subjected to Amber 99 energy minimization until the RMS of the conjugate gradient was 0.05 kcal ·mol^−1^·Å^−1^. Reduced units were used with a time step of 0.001 ps, and the simulation was performed until the potential energy U of the atomic system and kinetic energy K of the atoms stabilized. Default values were applied for other parameters. The resulting models were evaluated by PROCHECK [23] and VERIFY-3D [24] for geometry.

WT and nine mutants of DKR were chosen as targets for docking-based virtual screening. Receptor files, ligands and docking parameter files were prepared using MOE. The X-ray crystal structure of WT-DKR (PDB: 4E13) containing two NAD molecules was used. All hydrogen atoms and partial charges were added to the protein using Protonate 3D. The energy of the DKR-NAD^+^ complex was minimized with an energy minimization algorithm that uses the Amber 99 force field. This energy-minimized structure was used as a template for virtual screenings. The binding site was defined as a sphere encompassing protein residues within 4.5 Å of S122-H143-N146-N194, and was followed by restoration through London dG. Ligand placements were refined again by the Amber 99 force field. Default values were applied for other parameters, and 20 genetic algorithm runs were performed for each docking. Finally, a three-dimensional protein-substrate binding model was generated using MOE-2009. Ligand conformation was evaluated based on the S score, which measures interactions. Compounds showed different binding modes, and those with the lowest S scores were chosen for evaluation.

## Results

### Mutagenesis with canonical amino acid substitutions at position 222 and product analysis

WT-DKR reduces 2-chloro-1-phenylethanone to its corresponding alcohol 1-hydroxy-2-chloro-phenylethane with an *Re* face preference, while a Phe substitution at Trp222 inverted the enantiotope selectivity from *Re*- to *Si*- (Figure 1). This result suggests that substitution of Trp222 by amino acids with smaller side chains would reverse the fit of this substrate, which allows the cofactor to deliver its hydride to the *Si* face of the ketone, rather than the *Re* face. Based on the molecular volume and hydrophobicity of the twenty canonical amino acids, we chose hydrophobic amino acids including Val, Leu, Met, and Tyr to replace Trp in DKR mutants with smaller side chains at residue 222. Primers used in the mutagenesis are shown in Table 1. As anticipated, all mutants with smaller side chains exhibited an *Si*-preference producing S-alcohols (Table 2). Although W222V, W222L and W222M showed enantiomeric excess (*e.e.*) values that were comparable to W222F, W222Y showed an *e.e.* that was increased by 6.2-fold compared with W222F. Due to the small difference in molecular volume between Phe and Tyr, this significant and unusual increase of enantiopreference might be the result of increased polarity and additional H-bonding between the hydroxyl group of Tyr and the residue(s) in active center. Nevertheless, compared to WT-DKR, the results confirm that the smaller size of the side chain did affect the enantiotope preference and thus there could indeed be a correlation between the side chain and enantiopreference.

**Table 1:** Primers used for mutagenesis.

Mutant	Nucleotide sequences (5′- 3′)	Codon change
WT	ATAGGATCCGATGACCGGCATCACGAATG a	_
	GCGAAGCTTTCAGTACCGGTAGAAGCCCT b	
W222V	CAAGACGGTACGCATCGGCACGGGC a	TGG→GTA
	GCCCGTGCCGATGCGTACCGTCTTG b	
W222F	CAAGACGTTTCGCATCGGCACGGGC a	TGG→TTT
	GCCCGTGCCGATGCGAAACGTCTTG b	
W222L	CAAGACGCTGCGCATCGGCACGGGC a	TGG→CTG
	GCCCGTGCCGATGCGCAGCGTCTTG b	
W222M	CAAGACGATGCGCATCGGCACGGGC a	TGG→ATG
	GCCCGTGCCGATGCGCATCGTCTTG b	
W222Y	CAAGACGTATCGCATCGGCACGGGC a	TGG→TAT
	GCCCGTGCCGATGCGATACGTCTTG b	
W222TAG	CAAGACGTAGCGCATCGGCACGGGC a	TGG→TAG c
	GCCCGTGCCGATGCGCTACGTCTTG b	

a Sequence for forward primers.

b Sequence for reverse primers.

c Nucleotides for residue 222 were changed to TAG for UAA incorporation.

**Table 2:** Comparison of enantiopreference toward 2-chloro-1-phenylethanone for WT-DKR and nine DKR mutants.

Amino acid^a^	V	L	M	F	Y	CNF	W	MeOF	BiF	BuOF
Molecular Volume^b^	93.5	107.2	111.7	124.1	130.2	137.3	147.1	166.7	181.9	185.3
*e.e.* c (%)	9.8	3.8	1.4	5.4	33.3	7.1	9.1	10.5	29.7	33.7
Configuration	*S*	*S*	*S*	*S*	*S*	*S*	*R*	*R*	*R*	*R*

a Amino acid at residue 222.

b Molecular volume was from the PubMed compound database and refers to the van der Waals surface.

c Enantiomeric excess.

### Genetic incorporation of UAAs at DKR residue 222 and product analysis

**Figure 3:**
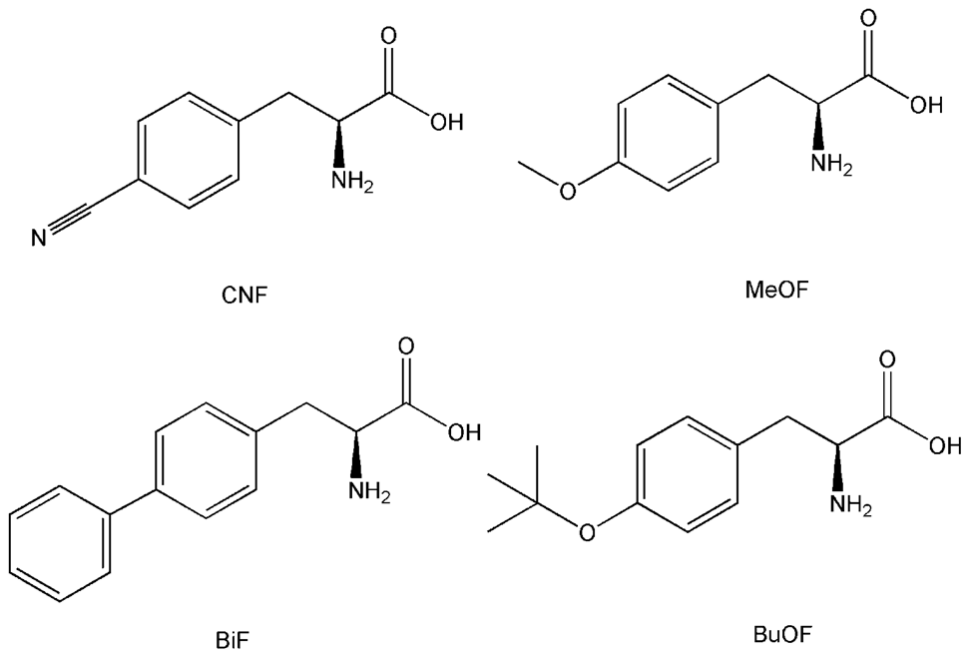
UAAs used for Trp222 substitution.

Since smaller amino acids at residue 222 in DKR caused preferential enantiotope inversion from *Re*- to *Si-*, if the molecular volume of the side chain is a major factor, substitution of Trp222 with bulky residues should result in a preference for the *Re*-face and production of *R*-alcohol, which is similar to WT, and the *e.e* would thus increase with side chain size. Trp has the largest side chain among the twenty canonical amino acids, so we genetically incorporated UAAs to increase the side chain size using an expanded genetic code. In this system, UAAs can be genetically incorporated into proteins using engineered orthogonal tRNA/aminoacyl-tRNA synthetase pairs. Briefly, an orthognal tRNA/synthetase pair evolved to be specific for the UAA should be expressed in the target cell together with the gene of interest. An amber stop codon UAG is introduced at the desired site for UAA insertion in the target gene. The orthogonal tRNA synthetase charges the UAA onto the cognate tRNA, which recognizes the UAG codon and then incorporates the UAA during translation [25]–[27]. Orthogonal *p*-cyanophenylalanine-specific aminoacyl-tRNA synthetase (pCNFRS), together with its cognate amber nonsense suppressor tRNA, can incorporate multiple UAAs with bulky side chains and the polyspecificity of pCNFRS allowed substitutions with multiple UAAs [28]. Four hydrophobic UAAs with various side chain sizes were chosen for incorporation into DKR at residue 222: 4-cyanophenylalanine (CNF); 4-methoxy-l-phenylalanine (MeOF); 4-phenyl-l-phenyalanine (BiF); and O-*tert*-butyl-l-tyrosine (BuOF) (Figure 3). We first introduced an amber condon at position 222 in the *dkr* gene in the pET22b(+) vector for UAA incorporation. Then the plasmid pCNFRSII-tRNA (Figure S1), which harbors two copies of pCNFRS and a copy of cognate suppressor tRNA, was constructed to express pCNFRS and tRNA. After co-transformation of *E. coli* BL21(DE3) cells with plasmid pCNFRSII-tRNA and pET22b(+)-DKR222TAG, four variants incorporating UAAs were expressed after IPTG induction. Mutant proteins were purified through two chromatographic steps as described previously [20]. Tryptic digestion and mass spectrometric analyses of variants verified the incorporation of UAAs (Table 3, Figure S2).

**Table 3:** MALDI-TOF/MS analyses of mutants containing unnatural amino acids (UAAs) after tryptic digestion.

UAA	Observed mass (Da)	Theoretical mass (Da)	Peptide fragment
CNF	1946.025	1946.023	LLVDGIADPETIDKTuR a
	1946.025	1946.023	LVDGIADPETIDKTuRI
MeOF	1424.777	1424.614	TIDKTuRIGTGAP
	1468.820	1468.819	DGIADPETIDKTu
	1667.501	1667.962	ADPETIDKTuRIGTG
BiF	947.473	947.472	ETIDKTu
	2158.142	2158.118	ETIDKTuRIGTGAPKGPFE
BuOF	1766.896	1766.917	VDGIADPETIDKTuR
	1894.975	1895.012	IADPETIDKTuRIGTGA

a u: unnatural amino acid corresponding to the listed UAA.

Similar to WT-DKR, 2-chloro-1-phenylethanone was reduced with *Re*-preference by the three mutants containing larger amino acids at residue 222. DKR Trp222 replacement by CNF, which contains a side chain that has a lower molecular volume than Trp, retained the *Si*-preference (Table 2). As indicated in Table 2, changes in enantiotope preference from *Si*- to *Re*- show positive correlation with increases in the molecular volume of the side chain. The most remarkable case is replacement of Trp222 with BuOF, which resulted in the largest switch of enantiopreference toward 2-chloro-1-phenylethanone (*e.e.* = 33.7%). Unlike smaller amino acid substitutions, increases in *Re*-preference correlated with side chain size increases when Trp222 was replaced by bulky residues, which supports the thinking that increased side chain bulk results in a more marked *Re*-preference.

### Biochemical properties of DKR mutants

We compared kinetic behaviors of mutants with that of WT-DKR (Table 4). Mutants W222Y, W222M, W222L and W222F showed notable (2.2–3.7-fold) increases in apparent *K_m_*. Mutant binding affinity for the substrate increased with bulkier UAAs. With the exception of the BiF substitution, the *k_cat_* values of all other variants decreased by varying degrees, with W222V, which has the smallest side chain, being only 20% that of WT-DKR. Meanwhile, the BiF mutant, with a biphenyl group at residue 222, showed a slight increase in *k_cat_* (0.34 to 0.50), which may result from a stronger BiF-dependent aromatic-aromatic interaction in the protein structure that enhances transition state stabilization. Overall, the catalytic efficiency (*k_cat_*/*K_m_*) of these mutants was largely affected by *K_m_* rather than *k_cat_*.

**Table 4:** Comparison of kinetic parameters and binding affinity of WT-DKR and nine mutants^a^^,^ b.

Amino acid^c^	*K_m_* (µM)	Vmax (µmol·min^−1^·mg^−1^)	*k_cat_* (S^−1^)^d^	*k*_cat_/*K*_m_ (M^−1^·S^−1^)×10^2^	*K*_d_ (µM)
V	301.9	0.14	0.07	2.32	506.5
L	3158.5	0.47	0.24	0.71	458.3
M	2801.6	0.44	0.22	0.79	457.1
F	4183.9	0.63	0.32	0.76	483.9
Y	2506.5	0.42	0.21	0.80	455.4
CNF	23.5	0.24	0.12	0.51	678.6
W	1129.4	0.68	0.34	3.01	459.9
MeOF	553.7	0.27	0.13	2.41	533.0
BiF	23.6	1.00	0.50	211.45	554.9
BuOF	5.1	0.20	0.10	194.55	636.9

a Initial velocity was obtained under conditions with varying 2-chloro-1-phenylethanone concentrations (0.1–3.4 mM) and a constant and saturating NADH concentration (0.15 mM). Data are the average of two measurements.

b Dissociation constants were determined using 80 µg protein as described in the Methods; *K*_d_ values were calculated by fitting the data to the quadratic equation. *K*_d_ values are the average of three measurements.

c Amino acids at residue 222.

d The *k_cat_* values were calculated based on the Michaelis-Menten model.

Fluorescence arising from aromatic residues can be used to probe the binding affinity between the substrate or cofactor NADH and the enzyme. To compare the catalytic properties of these variants, fluorescence quenching experiments were performed to compare binding affinities of WT and mutated DKR [24]. The variation trend of WT and mutant enzyme-substrate dissociation constant (*K*_d_) values was roughly consistent with *k_cat_*. The differences in *K*_d_ values were very minor and similar to WT values (Table 4). Thus, mutations at residue 222 did not disrupt enzyme-substrate binding, and the effects of these mutations on the DKR active site structure were modest.

### Structural evaluation of DKR mutants

To examine the role of Trp222 in enantiopreference, we modeled WT-DKR as well as nine DKR mutants complexed with NADH to compare structural differences. Models for the nine mutants were constructed based on the crystal structure of WT-DKR [19], as described in Methods. Ramachandran plot statistics of muatnt models were evaluated using the PROCHECK program. More than 99% of the dihedral angles of all residues in each mutant were located either in the most favored or in additionally allowed regions (Table S1). The VERIFY-3D score indicated good compatibility of the atomic model (3D) with the amino acid sequence (1D) (Table S1).

**Figure 4:**
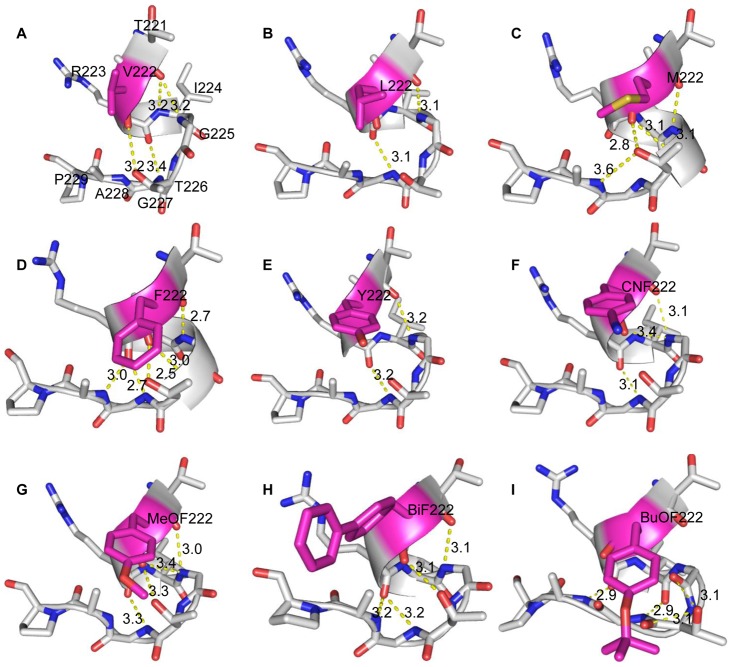
Helical conformation disruption by mutations at residue 222. Residues located at the α-helix 12 terminus are displayed as light gray sticks. Mutated residues are highlighted in magenta.

With the exception of BuOF (RMSD = 0.99 Å), an overlay between the structures of nine mutants and WT-DKR revealed a notable change in backbone architecture (RMSD>1.0 Å) (Table S2). The RMSD from individual residues indicated that remarkable changes occurred on α-helix 12 (RMSD>2 Å), where Trp222 is located (Figure S3). Hydrophobic interactions between residues showed disturbed helical conformations in the mutated proteins (Figure 4). The α-helix 12 in WT-DKR is a compact structure that ends at T226 (Figure 2B). For canonical amino acid substitutions, α-helix 12 terminated before T226, as was seen for the V, L, M and Y mutations (Figure 4A–C, E). For W222CNF, hydrogen bonding with T226 was abolished while hydrogen bond interactions with G225 and the end of the helix at I224 (Figure 4F) were constant, which was similar to the F222 and G225 interaction seen for the W222F DKR mutant (Figure 4D). When residue 222 was substituted with bulky UAAs, complicated structural changes resulted. W222MeOF (C = O) formed hydrogen bonds with T226 (O–H) and G225 (N–H) simultaneously, leading to a flat loop at the end of the helix (Figure 4G). In W222BiF, no hydrogen bonding between BiF222 and T226 occurred, but a new hydrogen bond formed between R223 (C = O) and A228 (N–H) for a tighter loop (Figure 4H). This tight loop was also observed for W222BuOF, in which multiple hydrogen bonds were formed between R223 (C = O) and A228 (N–H), between I224 (C = O) and G227 (N–H) and between T226 (O–H) and G225 (N–H) (Figure 4I). Thus, significant changes caused by residue 222 mutations resulted in a loop at the terminus of α-helix 12.

**Figure 5:**
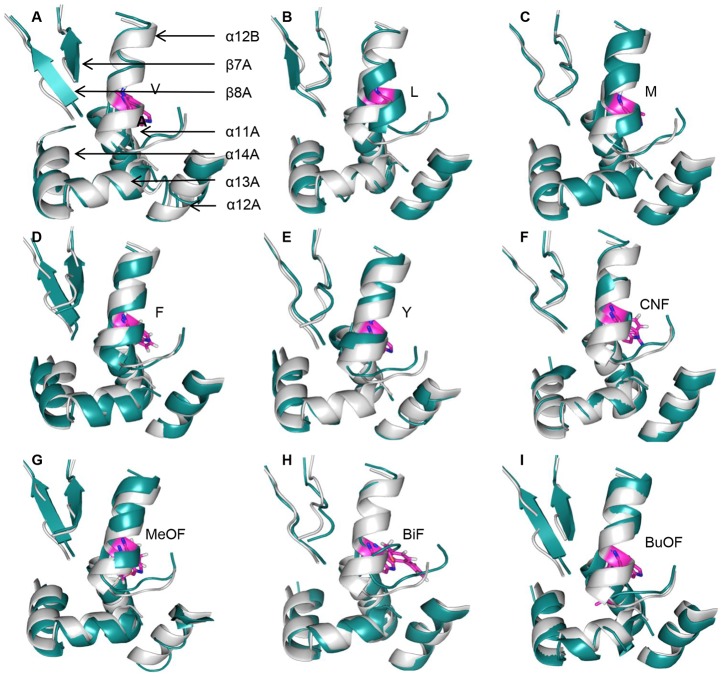
Comparison of the hydrophobic pocket for the nine DKR mutants and WT-DKR. In mutants containing V, L, M, F, Y and CNF at position 222, the α-helix 14 in DKR subunit A was pulled away from α-helix 12 in subunit B, and α-helix 11 in subunit A yielded a wider entrance (A, B, C, D, E, F). For MeOF, BiF, and BuOF, α-helix 14 in subunit A was closer to α-helix 11, consequently forming a tighter entrance for the substrate (G, H, I). Amino acids at residue 222 in subunit B are shown as magenta sticks. WT-DKR and mutants are colored light gray and deep teal, respectively.

Even though a marked change in α-helix 12 was observed in the mutants, this helix does not directly interact with the active center. A structural overlay between WT-DKR and the nine mutants was used to determine how the mutations might affect enzymatic enantiopreference (Figure 5). Remarkable differences were also seen in α-helix 14, which, together with α-helices 13 and β-strands 7 and 8 in subunit A and α-helix 12 in subunit B of the WT-DKR homodimer, form a substrate entrance channel to the active center (Figure 2A). Similar changes were observed in the RMSD (Figure S3). After replacing W222 by V, L, M, F, Y and CNF, the α-helix 14 of subunit A pulled away from α-helix 12 in subunit B, and α-helix 11 in subunit A formed a wider entrance to the active center. For MeOF, BiF and BuOF, α-helix 14 in subunit A moved closer to α-helix 11, which consequently formed a tighter entrance through which the substrate must pass.

In an attempt to rationalize these observations, we used computational docking to investigate the effects of these mutations on the binding of 2-chloro-1-phenylethanone to DKR variants. When WT-DKR and nine mutants were docked with the substrate, two opposing binding modes that produced respective alcohol enantiomers were found (Figure S4). Active site residues involved in substrate binding served as proton donors for substrate catalysis (Figure 5). Figure S5 shows the binding interactions of the substrate with each mutant. Docking scores with binding free energies (MM/GBVI) for the wild type and mutant proteins were in accordance with the respective enantiotope preference results (Table 5). The binding free energies of the pro-(*S*)-configuration binding mode were lower for V, L, M, F, Y and CNF substitutions, whereas WT and substitutions of MeoF, BiF and BuOF favored the pro-(*R*)-configuration binding mode. However, the docking score differences between each mode were not obvious, indicating that the enantiotope preference depends strongly on Trp222 substitution instead of the orientational preference for the active site itself.

**Table 5:** Docking score and binding free energy of 2-chloro-1-phenylethanone with different DKR mutants^a^.

Mutant	MM/GBVI^b^	Proton donor	Configuration
W222V	−6.1611	Thr243	*S*
	−5.5273	Thr242	*R*
W222L	−7.9193	Lys100	*S*
	−7.6216	Asn246	*R*
W222M	−9.5703	Glu95	*S*
	−7.5490	Glu95	*R*
W222F	−7.3150	Asn120	*S*
	−7.0301	Ser122	*R*
W222Y	−7.2933	Thr242	*S*
	−5.9048	NAD	*R*
W222CNF	−10.6496	Glu95	*S*
	−9.4981	Thr242	*R*
WT	−4.3826	Tyr245	*R*
	−3.8344	Asn246	*S*
W222MeOF	−7.3316	Lys100	*R*
	−4.4687	Lys100,Ser121	*S*
W222BiF	−9.4675	Asn246	*R*
	−7.6707	Asn246	*S*
W222BuOF	−8.2137	Asn246	*R*
	−8.1130	Asn146	*S*

a All data were calculated with Dock tools using Molecular Operating Environment (MOE2009; Chemical Computing Group Inc., Montreal, Canada).

b The binding free energy is the lowest in all obtained docking modes with two enantiopreferences.

**Figure 6:**
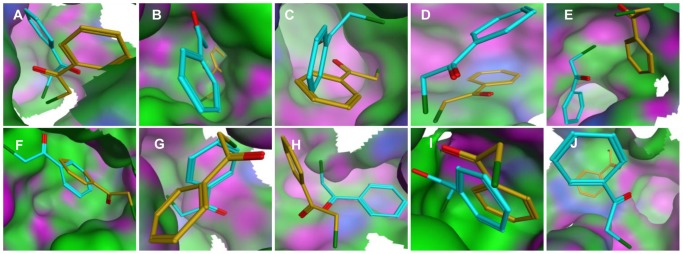
Substrate binding modes in the active center of WT and mutant DKR. For *Si*-preference enzymes (A, B, C, D, E, F), substrate orientations to produce different enantiomers randomly exist in the binding pocket. For *Re*-preference enzymes (G, H, I, J), a substrate that yields an *S*-enantiomer (yellow) adopts a “phenyl group first” position formed a pro-(*S*)-configuration, while the substrate (cyan) prefers the mode of “chloride first” and a pro-(*R*)-configuration. The substrate orientated in the pro-(*S*)-configuration is colored gold, and the substrate orientated in the pro-(*R*)-configuration is cyan. (A) W222V; (B) W222L; (C) W222M; (D) W222F; (E) W222Y; (F) W222CNF; (G) WT-DKR; (H) W222MeOF; (I) W222BiF; (J) W222BuOF.

Subsequently, substrate binding orientation with respect to the active site in each protein was analyzed. For the *Si*-preference mutants, substrate orientation was asymmetric and disordered (Figure 6A–F), due to the fact that the mutants containing smaller residues at residue 222 create a large entrance into the hydrophobic pocket that in turn results in a looser hydrophobic core. This wider form allows substrates to enter the active center with a flexible orientation without steric hindrance. In contrast, substrate orientations fall into a regular binding pattern for the four mutants having bulky amino acid substitutions. In the *Si*-preference binding mode, the subtrate prefers to enter the active center with the phenyl group first, whereas in the *Re*-preference binding mode the chloroacetyl group faces inside (Figure 6G–J). The orientation that the 2-chloro-1-phenylethanone adopts in the two different binding modes is consistent with side chain size changes.

## Discussion

Since the development of directed evolution, numerous efforts have been devoted to invert the enantiotope preference of enzymes with a number of mutants that induce inverted enantioselectivity in various enzymes having been produced. Currently, there are two explanations for how enantiotope selectivity could be inverted [29]: a) the position and orientation of active site residues exhibit distinct enantioselectivity with cooperative and collective changes of multiple residues occurring during catalysis, and at least one residue located close to the active center [8]; or b) the active site architecture is invariant, but the substrate binds to the active site in different orientations. In the second circumstance, mutations could occur in residues that are within, near, or far from the active site. In most situations, only one or two mutations are required for enantioselective inversion, and these could impose additional hydrophobic interactions [30] or directly occupy or release the central space, which would force the substrate to bind with a specific orientation [31]. In the case of residues without direct substrate or active center contact, Tang *et al.* successfully constructed P450pyr monooxygenase variants containing one or two mutations that exhibited enhanced enantioselectivity. One mutation at position 100 located near the active site entrance could invert the enantioselectivity by altering the conformation of the helix containing Asn100 upon substrate binding [32]. Similarly, Trp222 is an obvious “hot spot” in DKR that serves a “gate-keeper” function wherein the molecular volume of this residue governs the entrance direction and subsequent binding orientation of the substrate. Although residue hydrophilicity and polarity impacts enantioselectivity, in this work they often produced limited effects that do not involve inversion. Except for Tyr, the amino acids chosen for our study are hydrophobic and minimize the hydrophilic interactions between residue 222 and other residue side chains. Therefore, our results strongly support the latter hypothesis mentioned above that the “gate-keeper” residue influences steric strain on the substrate in the active site.

Generally, mutants exhibiting inverted enantiopreference and high activity usually contain multiple mutations with different combinations of mutations at various residues having distinct functions [8]. The residues responsible for either increases or decreases in catalytic activity are commonly located in the active cavity, and directly interact with the substrate or play a critical role during catalysis. Residues that determine enantiopreference of an enzyme are nearly always located either near the cavity entrance or a considerable distance away from the active center where they serve as “space holders”. Not surprisingly, the DKR mutants in the present study showed no enhanced activity. For further enhancements of activity, additional mutations at residues related to the active center are required. In our study the substrate itself may have contributed to this effect because of the two highly different moieties present in 2-chloro-1-phenylethanone: a larger phenyl group and smaller chlorine-substituted alkane. This structural asymmetry present in the substrate may highlight the importance of a particular residue that has a “gate keeper” function and can influence enantiotope preference. This possibility is consistent with a report on a ω-transaminase by Cassimjee *et al.*, in which a larger hydrophobic substrate binding pocket favors a phenyl group to produce an *S*-configuration [33].

Genetic incorporation of UAAs is a powerful tool to increase the structural diversity of proteins [34]. In the present study, we introduced four UAAs into DKR, which allowed us to probe the role of Trp222 and expand the utility of UAAs to study the relationship between amino acid residues and structural and functional changes. Although we incorporated only bulky UAAs into the enzyme here, other UAAs with boronate, azido, keto- and nitro- functional groups may invert enantioselectivity more efficiently through stronger interactions with some important amino acid residues that contribute to the catalysis.

The enantioselective reduction of prochiral ketones is useful in organic synthesis for producing chiral intermediates. Our work indicated that DKR catalyzes a series of ketone substrates with high efficiency and selectivity [15]. Therefore, it is conceivable that if important residues like Trp222 are replaced, opposite alcohol enantiomers from various ketone substrates can be obtained from different mutants of the enzyme.

In conclusion, we demonstrated that steric hindrance is a decisive factor for enantiotope preference when the mutation site is located relatively far from the active center. Meanwhile, inversion of enantiotope preference is caused by a binding pocket shape change to determine the structure of the substrate entrance channel and substrate binding orientation. The present study provides new insights into the role of a particular residue to determine enantiotope preference and will further facilitate *de novo* design of novel enzymes and molecular engineering of existing enzymes.

## Supporting Information

Figure S1 **Plasmid pEVOL-pCNFRSII for incorporation of UAAs.** The plasmid contains two copies of pCNFRS (expressed under the control of an *ara*BAD promoter) and a copy of a suppressor tRNA (expressed under the control of an *Ipp* promoter).
(DOC)

Figure S2 **MALDI-TOF/MS analysis of purified mutants with UAAs at residue 222 after tryptic digestion.** (A) CNF substitution; (B) MeOF substitution; (C) BiF substitution; (D) BuOF substitution.
(DOC)

Figure S3 **RMSD between WT-DKR and nine DKR mutants.** The RMSD by residue plot shows the residue-by-residue quality of superposition. For each alignment column used during the superposition, the RMSD value is represented by a vertical bar. Poor RMSD values are highlighted by dotted red horizontal lines with a 2.0 Å cutoff. Residue pairs above this line indicate obvious change. Residues marked by the pink line are located in α-helix 12. Residues located in α-helix 14 are marked by a blue line.
(DOC)

Figure S4 **Comparison of models for enzymatic catalysis. A hydride attack from two opposite orientations produces respective alcohol enantiomers.** Substrate 2-chloro-1-phenylethanone is shown as a teal sphere. Residues that serve as proton donors are highlighted in magenta. (A1) Pro-(*S*)-configurations in W222V; (A2) Pro-(*R*)-configurations in W222V; (B1) Pro-(*S*)-configurations in W222L; (B2) Pro-(*R*)-configurations in W222L; (C1) Pro-(*S*)-configurations in W222M; (C2) Pro-(*R*)-configurations in W222M; (D1) Pro-(*S*)-configurations in W222F; (D2) Pro-(*R*)-configurations in W222F; (E1) Pro-(*S*)-configurations in W222Y; (E2) Pro-(*R*)-configurations in W222Y; (F1) Pro-(*S*)-configurations in CNF; (F2) Pro-(*R*)-configurations in CNF; (G1) Pro-(*R*)-configurations in WT; (G2) Pro-(*S*)-configurations in WT; (H1) Pro-(*R*)-configurations in MeOF; (H2) Pro-(*S*)-configurations in WeOF; (I1) Pro-(*R*)-configurations in BiF; (I2) Pro-(*S*)-configurations in BiF; (J1) Pro-(*R*)-configurations in BuOF; (J2) Pro-(*S*)-configurations in BuOF.
(DOC)

Figure S5 **Binding interactions of 2-chloro-1-phenylethanone with WT-DKR and DKR mutants.** (A1) Substrate was attached from the *Si* face in W222V; (A2) Substrate was attached from the *Re* face in W222V; (B1) Substrate was attached from the *Si* face in W222L; (B2) Substrate was attached from the *Re* face in W222L; (C1) Substrate was attached from the *Si* face in W222M; (C2) Substrate was attached from the *Re* face in W222M; (D1) Substrate was attached from the *Si* face in W222F; (D2) Substrate was attached from the *Re* face in W222F; (E1) Substrate was attached from the *Si* face in W222Y; (E2) Substrate was attached from the *Re* face in W222Y; (F1) Substrate was attached from the *Si* face in CNF; (F2) Substrate was attached from the *Re* face in CNF; (G1) Substrate was attached from the *Re* face in WT; (G2) Substrate was attached from the *Si* face in WT; (H1) Substrate was attached from the *Re* face in MeOF; (H2) Substrate was attached from the *Si* face in MeOF; (I1) Substrate was attached from the *Re* face in BiF; (I2) Substrate was attached from the *Si* face in BiF; (J1)Substrate was attached from the *Re* face in BuOF; (J2) Substrate was attached from the *Si* face in BuOF.
(DOC)

Table S1 Stereochemical quality and model evaluation of WT-DKR and DKR mutants.
(DOC)

Table S2 Root mean square deviations (RMSDs, Å) of WT-DKR and mutants^a^.
(DOC)
