# Supplementary material for: The “Gate Keeper” Role of Trp222 Determines the Enantiopreference of Diketoreductase toward 2-Chloro-1-Phenylethanone
Source: PLoS One. 2014 Jul 29;9(7):e103792. doi: 10.1371/journal.pone.0103792 (PMC4114983; doi:10.1371/journal.pone.0103792)
Supplement: Figure S1 — Plasmid pEVOL-pCNFRSII for incorporation of UAAs. The plasmid contains two copies of pCNFRS (expressed under the control of an araBAD promoter) and a copy of a suppressor tRNA (expressed under the control of an Ipp promoter). (DOC) [file pone.0103792.s001.doc]

**Supporting information**

**
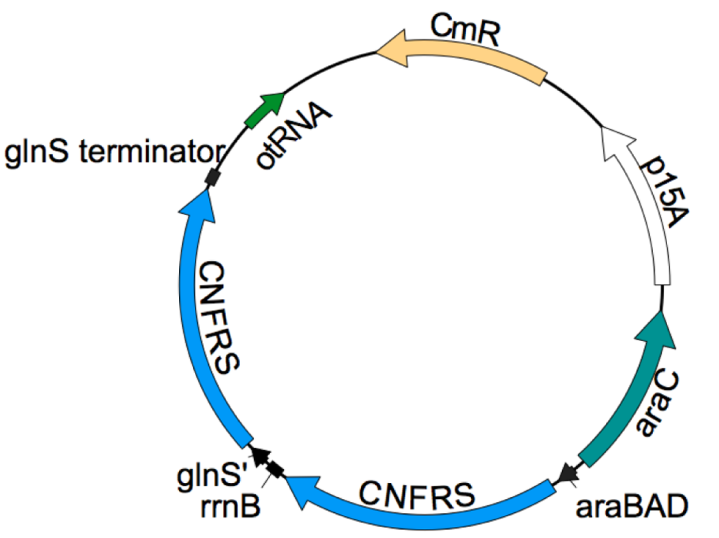
**

**Figure S1 Plasmid pCNFRSII-tRNA** **for incorporation of UAAs.** The plasmid contains two copies of pCNFRS (expressed under the control of an *ara*BAD promoter) and a copy of a suppressor tRNA (expressed under the control of an *Ipp* promoter).
