# Supplementary material for: The “Gate Keeper” Role of Trp222 Determines the Enantiopreference of Diketoreductase toward 2-Chloro-1-Phenylethanone
Source: PLoS One. 2014 Jul 29;9(7):e103792. doi: 10.1371/journal.pone.0103792 (PMC4114983; doi:10.1371/journal.pone.0103792)
Supplement: Figure S2 — MALDI-TOF/MS analysis of purified mutants with UAAs at residue 222 after tryptic digestion. (A) CNF substitution; (B) MeOF substitution; (C) BiF substitution; (D) BuOF substitution. (DOC) [file pone.0103792.s002.doc]

**Supporting information**

A


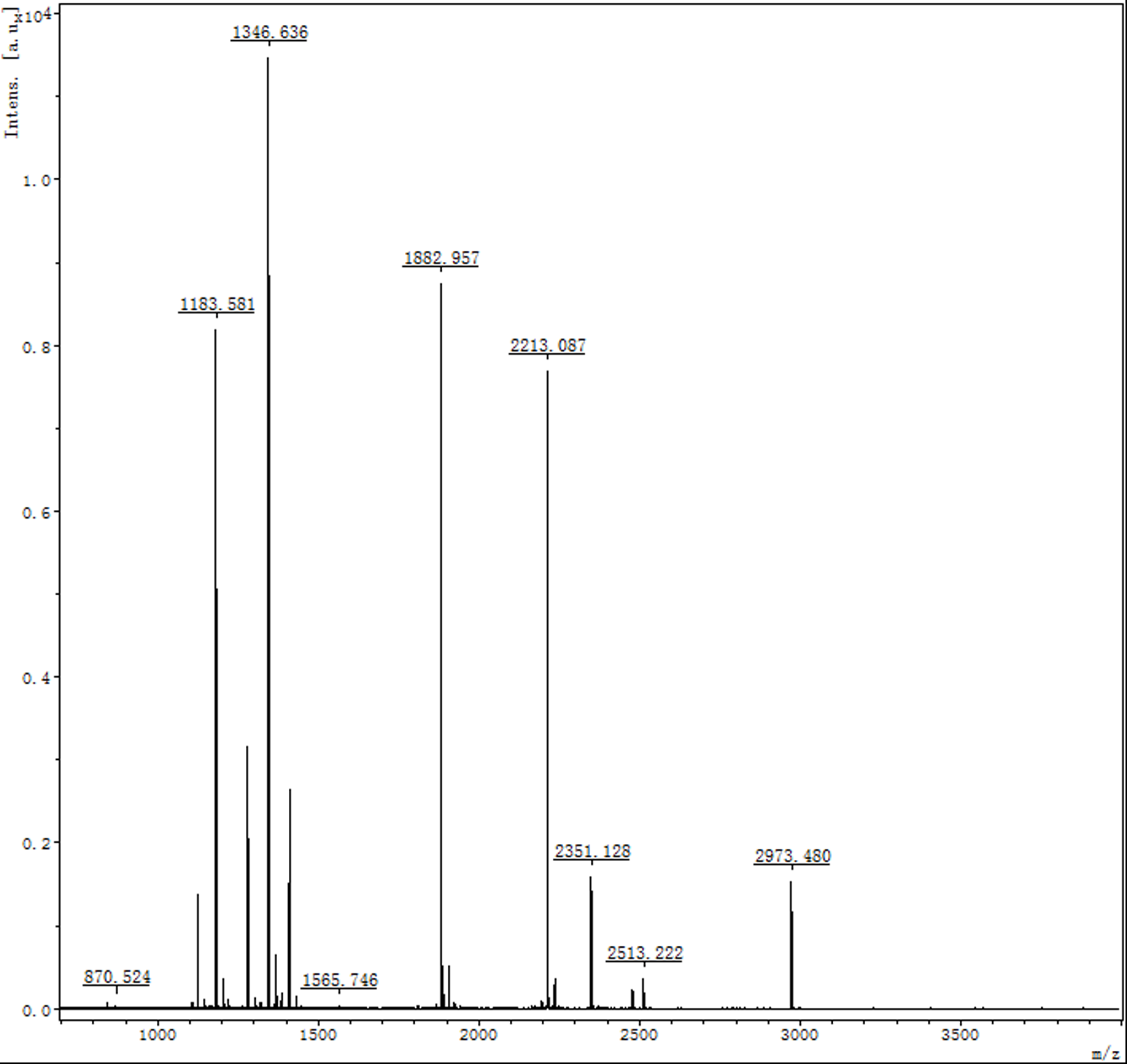


B


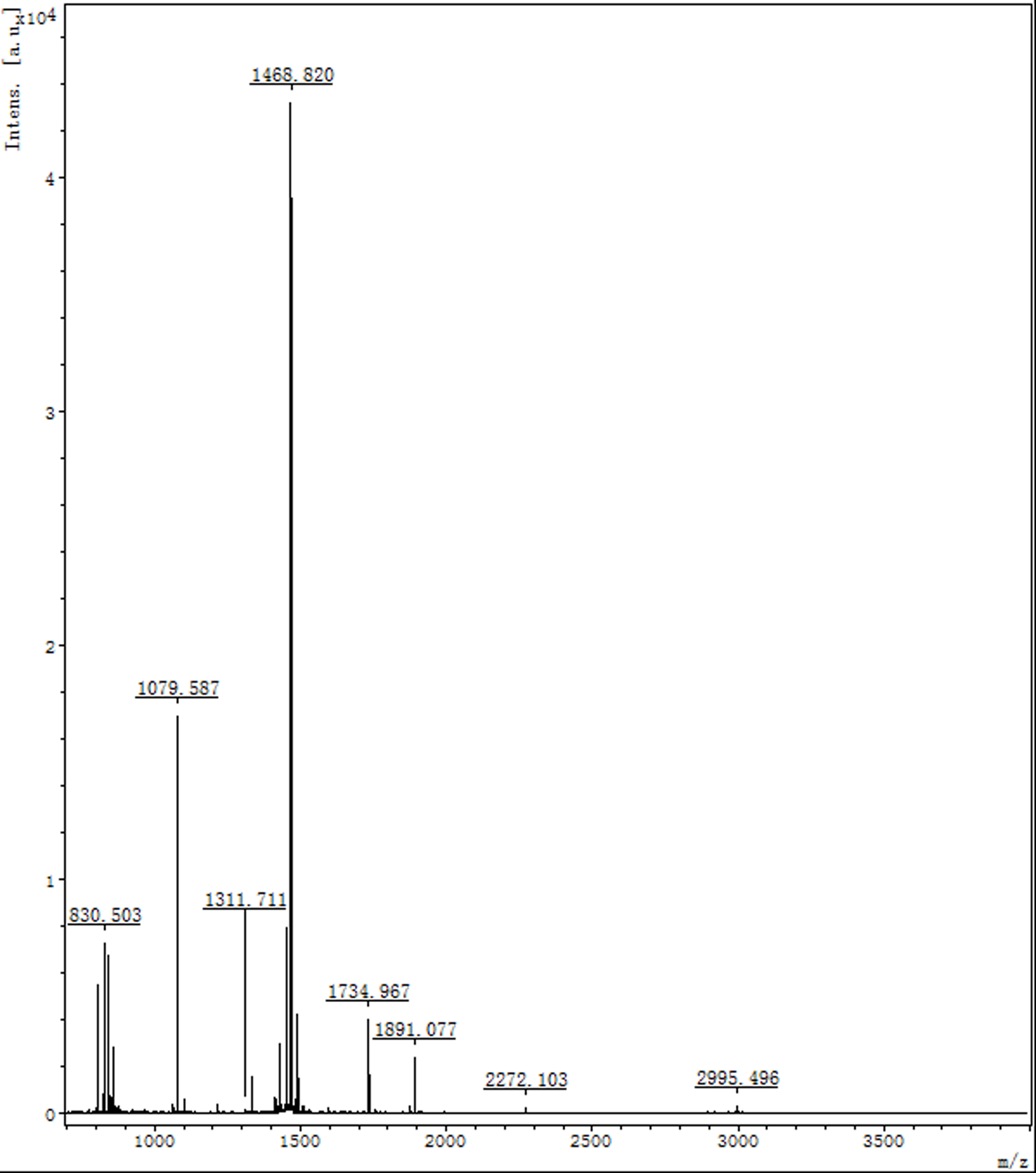


C


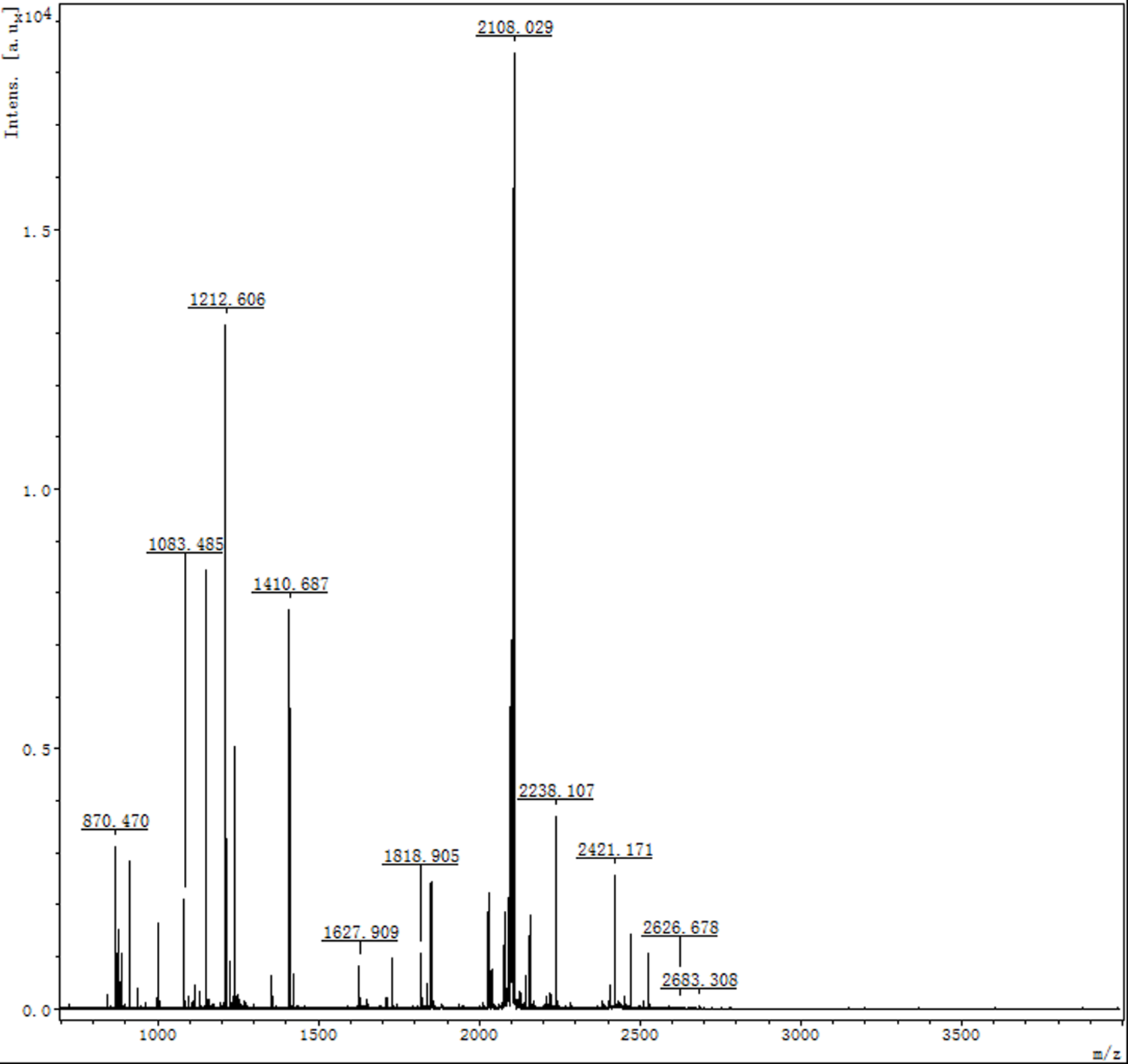


D


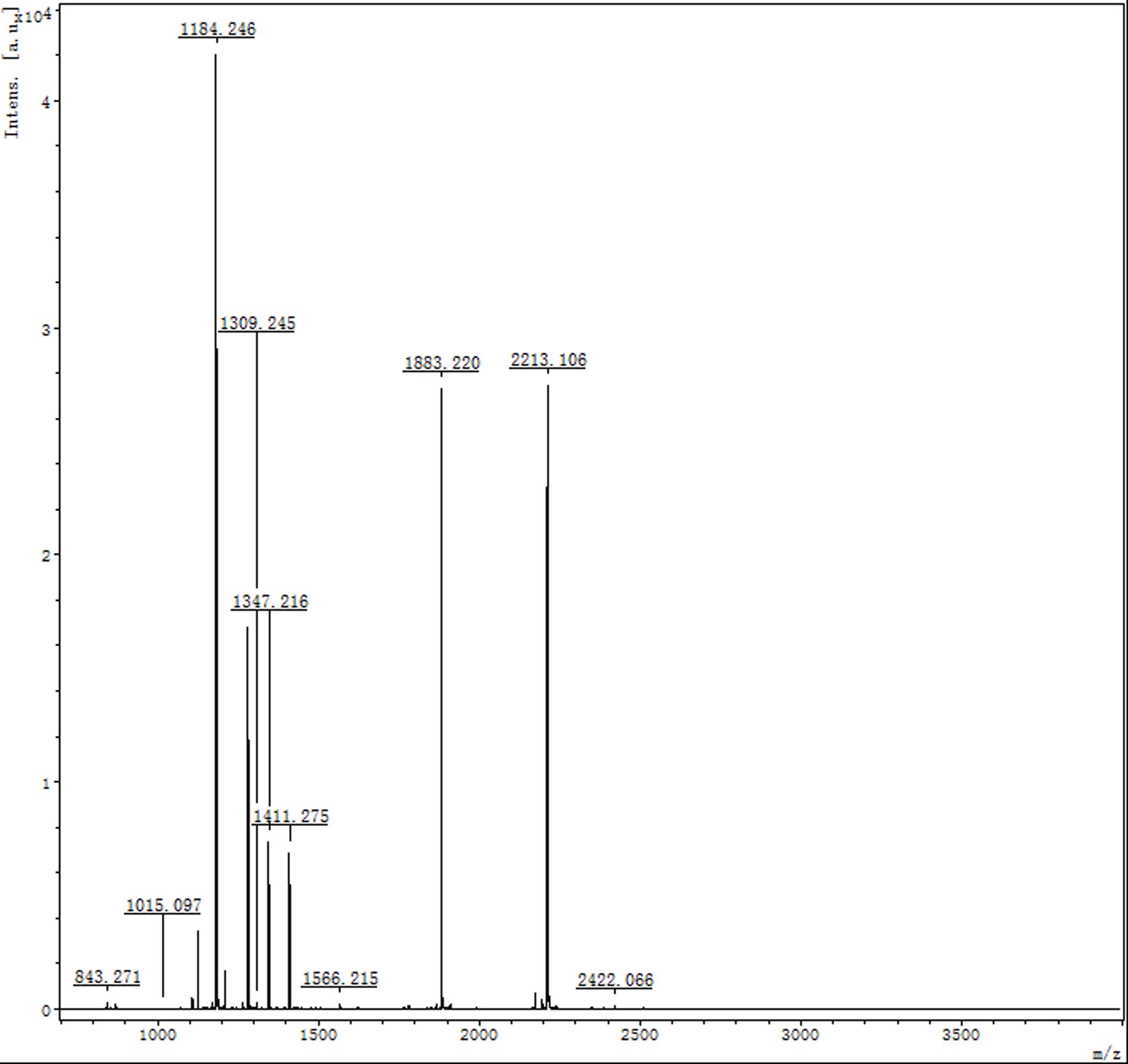


**Figure S2**. **MALDI-TOF/MS analysis of purified mutants wtih unnatural amino acids at residue 222 after tryptic digestion.** (A) substitution by CNF; (B) substitution by MeOF; (C) substitution by BiF; (D) substitution by BuOF**.**
