# Supplementary material for: The “Gate Keeper” Role of Trp222 Determines the Enantiopreference of Diketoreductase toward 2-Chloro-1-Phenylethanone
Source: PLoS One. 2014 Jul 29;9(7):e103792. doi: 10.1371/journal.pone.0103792 (PMC4114983; doi:10.1371/journal.pone.0103792)
Supplement: Figure S3 — RMSD between WT-DKR and nine DKR mutants. The RMSD by residue plot shows the residue-by-residue quality of superposition. For each alignment column used during the superposition, the RMSD value is represented by a vertical bar. Poor RMSD values are highlighted by dotted red horizontal lines with a 2.0 Å cutoff. Residue pairs above this line indicate obvious change. Residues marked by the pink line are located in α-helix 12. Residues located in α-helix 14 are marked by a blue line. (DOC) [file pone.0103792.s003.doc]

**Supporting information**

**
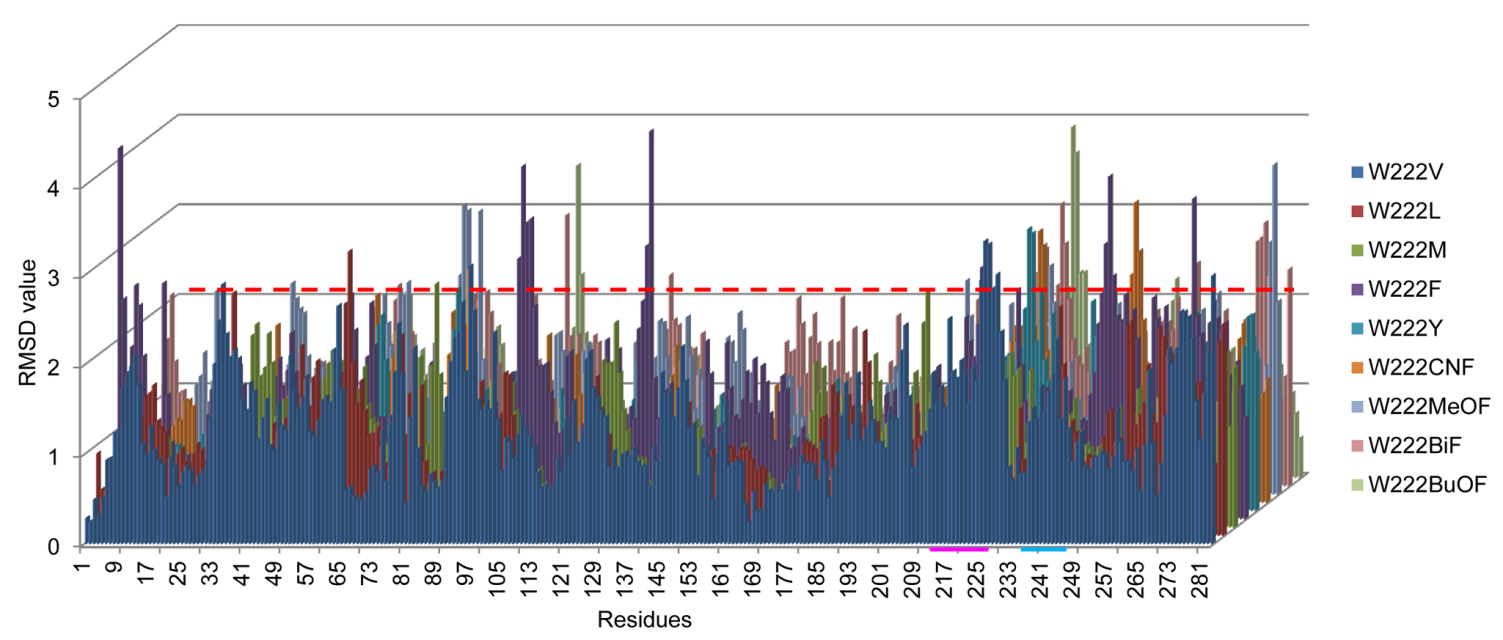
**

**Figure S3.** **RMSD between WT-DKR and nine DKR mutants.** The RMSD by residue plot shows the residue-by-residue quality of superposition. For each alignment column used during the superposition, the RMSD value is represented by a vertical bar. Poor RMSD values are highlighted by dotted red horizontal lines with a 2.0 Å cutoff. Residue pairs above this line indicate obvious change. Residues marked by the pink line are located in α-helix 12. Residues located in α-helix 14 are marked by a blue line.
