# Supplementary material for: The “Gate Keeper” Role of Trp222 Determines the Enantiopreference of Diketoreductase toward 2-Chloro-1-Phenylethanone
Source: PLoS One. 2014 Jul 29;9(7):e103792. doi: 10.1371/journal.pone.0103792 (PMC4114983; doi:10.1371/journal.pone.0103792)
Supplement: Figure S4 — Comparison of models for enzymatic catalysis. A hydride attack from two opposite orientations produces respective alcohol enantiomers. Substrate 2-chloro-1-phenylethanone is shown as a teal sphere. Residues that serve as proton donors are highlighted in magenta. (A1) Pro-(S)-configurations in W222V; (A2) Pro-(R)-configurations in W222V; (B1) Pro-(S)-configurations in W222L; (B2) Pro-(R)-configurations in W222L; (C1) Pro-(S)-configurations in W222M; (C2) Pro-(R)-configurations in W222M; (D1) Pro-(S)-configurations in W222F; (D2) Pro-(R)-configurations in W222F; (E1) Pro-(S)-configurations in W222Y; (E2) Pro-(R)-configurations in W222Y; (F1) Pro-(S)-configurations in CNF; (F2) Pro-(R)-configurations in CNF; (G1) Pro-(R)-configurations in WT; (G2) Pro-(S)-configurations in WT; (H1) Pro-(R)-configurations in MeOF; (H2) Pro-(S)-configurations in WeOF; (I1) Pro-(R)-configurations in BiF; (I2) Pro-(S)-configurations in BiF; (J1) Pro-(R)-configurations in BuOF; (J2) Pro-(S)-configurations in BuOF. (DOC) [file pone.0103792.s004.doc]

**Supporting information**

**
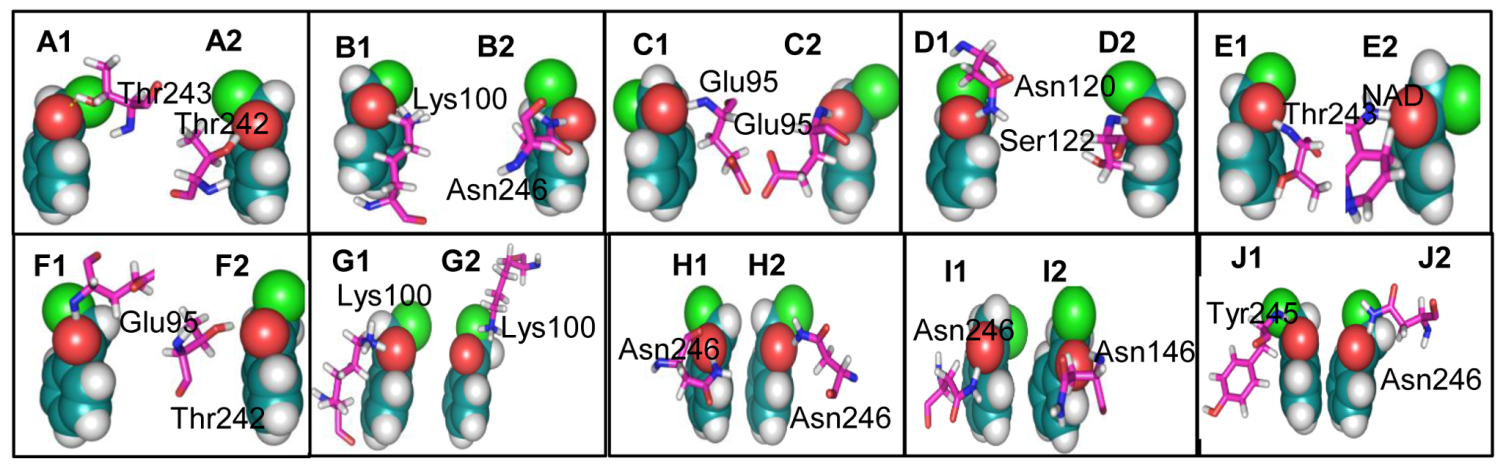
**

**Figure S4.** **Comparison of models for enzymatic catalysis. A hydride attack from two opposite orientations produces respective alcohol enantiomers.** Substrate 2-chloro-1-phenylethanone is shown as a teal sphere. Residues that serve as proton donors are highlighted in magenta. (A1) Pro-(*S*)-configurations in W222V; (A2) Pro-(*R*)-configurations in W222V; (B1) Pro-(*S*)-configurations in W222L; (B2) Pro-(*R*)-configurations in W222L; (C1) Pro-(*S*)-configurations in W222M; (C2) Pro-(*R*)-configurations in W222M; (D1) Pro-(*S*)-configurations in W222F; (D2) Pro-(*R*)-configurations in W222F; (E1) Pro-(*S*)-configurations in W222Y; (E2) Pro-(*R*)-configurations in W222Y; (F1) Pro-(*S*)-configurations in CNF; (F2) Pro-(*R*)-configurations in CNF; (G1) Pro-(*R*)-configurations in WT; (G2) Pro-(*S*)-configurations in WT; (H1) Pro-(*R*)-configurations in MeOF; (H2) Pro-(*S*)-configurations in WeOF; (I1) Pro-(*R*)-configurations in BiF; (I2) Pro-(*S*)-configurations in BiF; (J1) Pro-(*R*)-configurations in BuOF; (J2) Pro-(*S*)-configurations in BuOF.
