# Supplementary material for: The “Gate Keeper” Role of Trp222 Determines the Enantiopreference of Diketoreductase toward 2-Chloro-1-Phenylethanone
Source: PLoS One. 2014 Jul 29;9(7):e103792. doi: 10.1371/journal.pone.0103792 (PMC4114983; doi:10.1371/journal.pone.0103792)
Supplement: Figure S5 — Binding interactions of 2-chloro-1-phenylethanone with WT-DKR and DKR mutants. (A1) Substrate was attached from the Si face in W222V; (A2) Substrate was attached from the Re face in W222V; (B1) Substrate was attached from the Si face in W222L; (B2) Substrate was attached from the Re face in W222L; (C1) Substrate was attached from the Si face in W222M; (C2) Substrate was attached from the Re face in W222M; (D1) Substrate was attached from the Si face in W222F; (D2) Substrate was attached from the Re face in W222F; (E1) Substrate was attached from the Si face in W222Y; (E2) Substrate was attached from the Re face in W222Y; (F1) Substrate was attached from the Si face in CNF; (F2) Substrate was attached from the Re face in CNF; (G1) Substrate was attached from the Re face in WT; (G2) Substrate was attached from the Si face in WT; (H1) Substrate was attached from the Re face in MeOF; (H2) Substrate was attached from the Si face in MeOF; (I1) Substrate was attached from the Re face in BiF; (I2) Substrate was attached from the Si face in BiF; (J1)Substrate was attached from the Re face in BuOF; (J2) Substrate was attached from the Si face in BuOF. (DOC) [file pone.0103792.s005.doc]

**Supporting information**

**
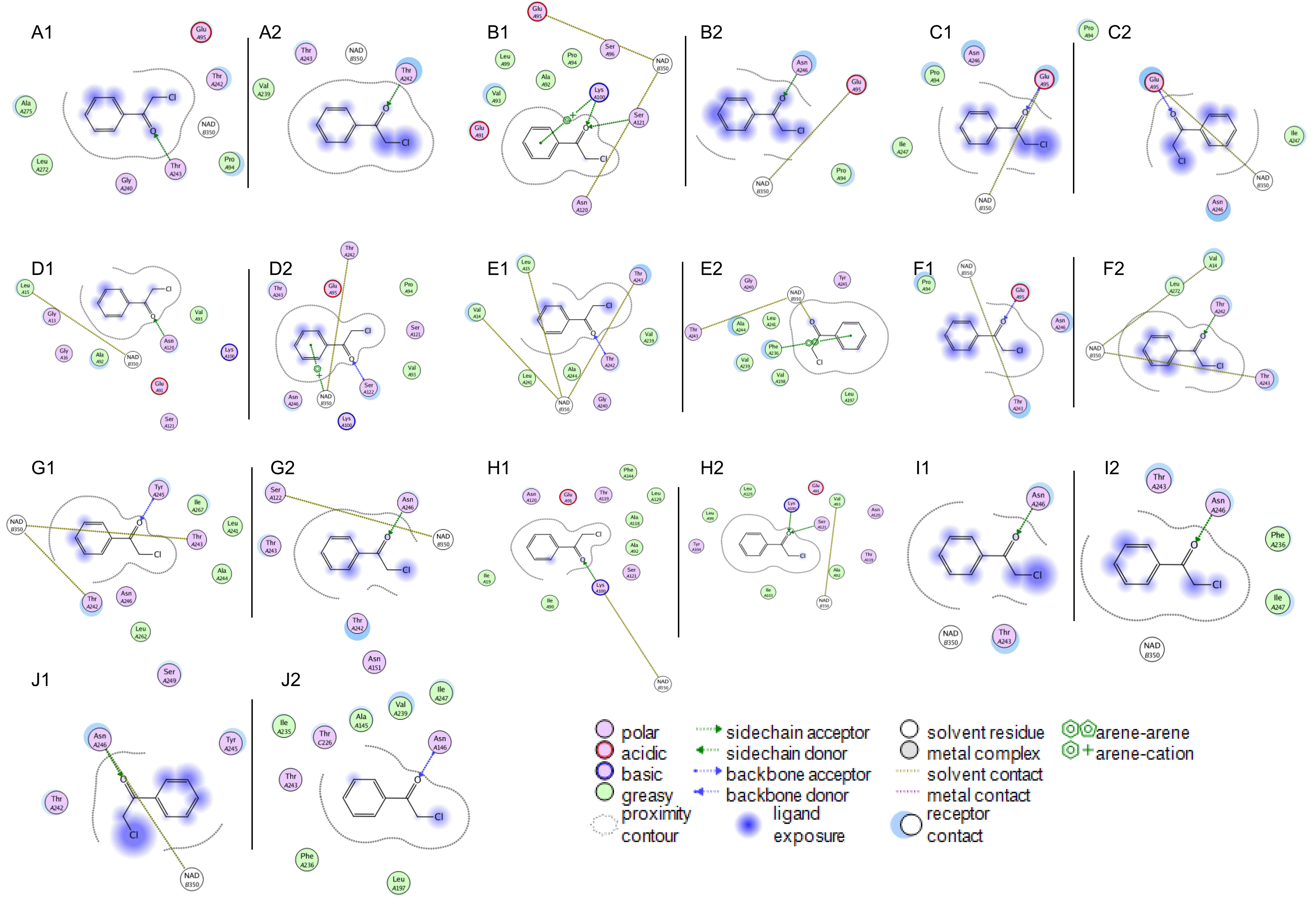
**

**Figure S5. Binding interactions of 2-chloro-1-phenylethanone with WT-DKR and DKR mutants.** (A1) Substrate was attached from the *Si* face in W222V; (A2) Substrate was attached from the *Re* face in W222V; (B1) Substrate was attached from the *Si* face in W222L; (B2) Substrate was attached from the *Re* face in W222L; (C1) Substrate was attached from the *Si* face in W222M; (C2) Substrate was attached from the *Re* face in W222M; (D1) Substrate was attached from the *Si* face in W222F; (D2) Substrate was attached from the *Re* face in W222F; (E1) Substrate was attached from the *Si* face in W222Y; (E2) Substrate was attached from the *Re* face in W222Y; (F1) Substrate was attached from the *Si* face in CNF; (F2) Substrate was attached from the *Re* face in CNF; (G1) Substrate was attached from the *Re* face in WT; (G2) Substrate was attached from the *Si* face in WT; (H1) Substrate was attached from the *Re* face in MeOF; (H2) Substrate was attached from the *Si* face in MeOF; (I1) Substrate was attached from the *Re* face in BiF; (I2) Substrate was attached from the *Si* face in BiF; (J1)Substrate was attached from the *Re* face in BuOF; (J2) Substrate was attached from the *Si* face in BuOF.
