# Supplementary material for: The “Gate Keeper” Role of Trp222 Determines the Enantiopreference of Diketoreductase toward 2-Chloro-1-Phenylethanone
Source: PLoS One. 2014 Jul 29;9(7):e103792. doi: 10.1371/journal.pone.0103792 (PMC4114983; doi:10.1371/journal.pone.0103792)
Supplement: Table S1 — Stereochemical quality and model evaluation of WT-DKR and DKR mutants. (DOC) [file pone.0103792.s006.doc]

**Supporting information**

**Table S1.** Stereochemical quality and model evaluation of WT-DKR and mutants.

| Predicted models | PROCHECK analysis showing residues at carious regions | | | | VERIFY 3D-1D score(%) ^a^ |
| --- | --- | --- | --- | --- | --- |
|  | Core (%) | Allow(%) | Generously(%) | Disallowed (%) |  |
| W222V | 79.7 | 16.6 | 3.7 | 0 | 98.59 |
| W222L | 78.5 | 18 | 3.5 | 0 | 98.94 |
| W222M | 79.5 | 17.8 | 2.7 | 0 | 99.30 |
| W222F | 86.5 | 12.5 | 0.8 | 0.2 | 100 |
| W222Y | 79.9 | 17 | 2.9 | 0.2 | 99.3 |
| sCNF | 79.1 | 16.5 | 4.3 | 0 | 98.94 |
| WT | 78.7 | 17.6 | 3.7 | 0 | 100 |
| sMeOF | 81.4 | 14.3 | 4.3 | 0 | 98.59 |
| sBiF | 77.9% | 18.6 | 3.5 | 0 | 96.48 |
| sBuOF | 75.4 | 22.7 | 1.4 | 0.4 | 97.54 |

^a^ Percentage of residues had an averaged 3D-1D score>0.2
