# Supplementary material for: The “Gate Keeper” Role of Trp222 Determines the Enantiopreference of Diketoreductase toward 2-Chloro-1-Phenylethanone
Source: PLoS One. 2014 Jul 29;9(7):e103792. doi: 10.1371/journal.pone.0103792 (PMC4114983; doi:10.1371/journal.pone.0103792)
Supplement: Table S2 — Root mean square deviations (RMSDs, Å) of WT-DKR and mutantsa. (DOC) [file pone.0103792.s007.doc]

**Supporting information**

**Table S2.** Root mean square deviations (RMSDs; Å) of WT-DKR and mutants^a^.

| Enzyme | W222V | W222L | W222M | W222F | W222Y | sCNF | sMeOF | sBiF | sBuOF |
| --- | --- | --- | --- | --- | --- | --- | --- | --- | --- |
| Average RMSD/Å | 1.48 | 1.17 | 1.08 | 1.53 | 1.08 | 1.09 | 1.31 | 1.28 | 0.99 |

^a^ RMSD values were calculated upon pairwise superposition of equivalent pairs of Cα atoms from a monomer by Superpose tools using MOE.
